# Reply to the Letter to the Editor: “Self-compassion in migraine: associations with depression, anxiety, and clinical characteristics” — methodological and statistical considerations

**DOI:** 10.1055/s-0046-1829167

**Published:** 2026-10-23

**Authors:** Ruken Simsekoglu, Zarife Tugce Yıldız, Nilay Garipbas

**Affiliations:** 1 Istanbul Göztepe Prof. Dr. Suleyman Yalcın City Hospital, Department of Neurology, IstanbulTurkey.; 2 Istanbul Göztepe Prof. Dr. Suleyman Yalcın City Hospital, Department of Psychology, IstanbulTurkey.

Dear Editor,

We sincerely thank the author for the interest shown in our article and for the thoughtful comments. We appreciate the opportunity to further clarify several methodological and statistical aspects of our study. 1 We concur that self-compassion in individuals with migraine is an important and emerging topic in neuropsychiatric research, and we hope that the following responses will help clarify the issues raised.

Given the exploratory nature of our study, and the application of strict inclusion and exclusion criteria, the sample size, particularly within the migraine subgroups, was relatively limited. Therefore, as acknowledged in the *Discussion* section, the findings from these subgroup analyses should be interpreted with appropriate caution. Furthermore, this limitation was explicitly addressed in the *Limitations* section, where we emphasized that future studies with larger and more representative migraine subgroups are needed to validate and extend our findings. 2

We acknowledge the author's point that the use of a structured diagnostic interview, such as the Structured Clinical Interview for DSM Disorders (SCID), would represent the gold standard for psychiatric assessment. However, all participants underwent comprehensive neurological evaluation by the same neurologist, and neuropsychological assessments were conducted by an experienced clinical psychologist before enrolment. In addition, participants with a history or current diagnosis of major psychiatric disorders were excluded based on clinical interviews and review of medical records. This approach is commonly employed in clinical research, and because the same assessment procedures were applied to both the migraine and control groups, we believe that the potential risk of assessment bias was minimized.

We would like to further clarify the rationale behind our regression analysis. The purpose of the regression model was not to identify clinical predictors within the migraine cohort. Rather, it was specifically designed to examine whether migraine status was independently associated with self-compassion after controlling for demographic variables, depression, and anxiety. Accordingly, migraine-related clinical characteristics such as disease duration, aura status, medication overuse, and pain severity were not included in the model, as these variables were not relevant to the primary research question and are applicable only to the migraine group. Furthermore, given the relatively small sample size of the clinical subgroups, we believe that constructing a multivariable model including these variables would have resulted in limited statistical power and potentially unstable estimates. Regarding the concern about multiple comparisons, Bonferroni correction was applied where appropriate for subgroup analyses. The remaining analyses were exploratory in nature and should be interpreted accordingly. Finally, we would also like to emphasize that the correlation analyses were intended to be exploratory, and the limitations related to the modest subgroup sample sizes were explicitly acknowledged and discussed in the *Discussion* and *Limitations* sections of our article. 1

We recognize that causal inferences cannot be drawn from a cross-sectional study. As stated in the *Discussion* section, our findings were interpreted as associative rather than causal, and we emphasized the need for longitudinal studies.

In conclusion, we sincerely thank the authors for their constructive comments. We are grateful for the opportunity to further explain the methodological and statistical rationale underlying our study. We hope that this exchange will contribute to a clearer interpretation of our findings and encourage further research on the relationship between self-compassion and migraine.
